# Lateralized Kinematics of Predation Behavior in a Lake Tanganyika Scale-Eating Cichlid Fish

**DOI:** 10.1371/journal.pone.0029272

**Published:** 2012-01-06

**Authors:** Yuichi Takeuchi, Michio Hori, Yoichi Oda

**Affiliations:** 1 Graduate School of Science, Nagoya University, Nagoya, Japan; 2 Department of Biological Science, Kyoto University, Kyoto, Japan; Institute of Marine Research, Norway

## Abstract

Behavioral lateralization has been documented in many vertebrates. The scale-eating cichlid fish *Perissodus microlepis* is well known for exhibiting lateral dimorphism in its mouth morphology and lateralized behavior in robbing scales from prey fish. A previous field study indicated that this mouth asymmetry closely correlates with the side on which prey is attacked, but details of this species' predation behavior have not been previously analyzed because of the rapidity of the movements. Here, we studied scale-eating behavior in cichlids in a tank through high-speed video monitoring and quantitative assessment of behavioral laterality and kinematics. The fish observed showed a clear bias toward striking on one side, which closely correlated with their asymmetric mouth morphologies. Furthermore, the maximum angular velocity and amplitude of body flexion were significantly larger during attacks on the preferred side compared to those on the nonpreferred side, permitting increased predation success. In contrast, no such lateral difference in movement elements was observed in acoustically evoked flexion during the escape response, which is similar to flexion during scale eating and suggests that they share a common motor control pathway. Thus the neuronal circuits controlling body flexion during scale eating may be functionally lateralized upstream of this common motor pathway.

## Introduction

Anatomical brain lateralization, e.g., the asymmetric organization of the left and right hemispheres, has been widely documented among vertebrates from fish to mammals [1], [2]. Brain lateralization may give rise to an increase in cognitive abilities, behavioral complexity, or behavioral laterality, leading to advantages in brain function (see [3] for a review). For example, chicks with lateralized brains were found to detect a raptor stimulus with shorter latency than were nonlateralized chicks [4], and lateralization enhances the ability of chicks to perform two tasks simultaneously [5]. A similar finding in killifish was also observed by Dadda & Bisazza [6]. Despite many documented examples of behavioral laterality in various organisms [1], [7], there is little direct evidence of the neuronal basis for behavioral laterality in vertebrates. The complexity of the neuronal basis and the subtlety of the differences in morphological asymmetry increase the difficulty of analysis. Thus, few attempts have been made to link lateralized behaviors to their underlying neuronal mechanisms.

A remarkable example of left–right asymmetry has been shown in the mouth morphology and corresponding scale-eating behavior of the Lake Tanganyikan cichlid fish known as the Perissodini [8]–[10]. These species forage on scales by attacking the left or right flank of prey fish using their asymmetric mouths [11], [12]. The Perissodini possess dental morphology and craniofacial asymmetry specially evolved for scale eating [13], [14]. Due to differential development and/or remodeling in the sides of the lower jawbone, their mouths open either leftward or rightward [14], [15]. That is, lefty fish whose left-side jawbone is longer than the right side have a mouth skewed toward the right, and the reverse is true for righty fish. Among the Perissodini tribe, *Perissodus microlepis* exhibit especially clear mouth asymmetry [8], [9]. Analysis of the stomach contents of scale-eating fish in the field indicates that lefties attack only the left sides of prey fish with their mouths bent to the right, and vice versa [9]. Therefore, the laterally dimorphic mouth of *P. microlepis* is considered to correspond closely to the side of attack. Furthermore, *P. microlepis* exhibits left–right differences in its cranial morphology which may also underlie lateralization in the speed and force of lower jaw rotation [14]. Takahashi et al. [12] described the interspecific difference of foraging behaviors between *P. microlepis* and another scale eater, *Perissodus straeleni*, from video camera recordings at low time resolution (30 frames/s). However, the predation motions themselves remain poorly understood due to their rapidity (less than 10 ms in duration), and an understanding of the neuronal mechanisms underlying this lateralized behavior is completely lacking.

The aims of the present study were, first, to clarify the laterality and kinematics of predation behavior of the scale-eating cichlid fish *Perissodus microlepis*, and second, to address the potential neuronal pathways for the lateralized behavior. Predation behavior was observed in a tank via a high-speed video camera and analyzed quantitatively. Lateralized behavior was observed in the movements made during attacks with a clear bias of kinematics for one particular striking side, which correlated closely with its asymmetric mouth morphology. The fast bending that occurred during prey strikes was similar to the C-bend that occurs during fast escapes, which is initiated by a single bilateral pair of giant reticulospinal neurons in the hindbrain, the Mauthner cells (M-cells) [16]–[18], through the firing of one of them activating spinal motoneurons and associated interneurons, allowing the contralateral trunk and tail muscles to contract simultaneously (see [19] for a review). The similarity of body flexion during scale-eating and escape behaviors indicates that possibility that there is also a similarity in the neuronal substrate underlying these two behaviors.

## Results

### Behavioral component of predation

*P. microlepis* exhibited predation behavior and removed scales from prey fish in a tank as in the field. We monitored the predation behavior of 20 scale eaters using a high-speed video camera (500 frames/s). Before approaching prey, a scale eater usually hid in a shady space and watched for prey at a distance of 21–310 mm (138±80 mm, mean ± SD, n = 43). It began pursuing the prey when the prey turned its back.

**Figure 1:**
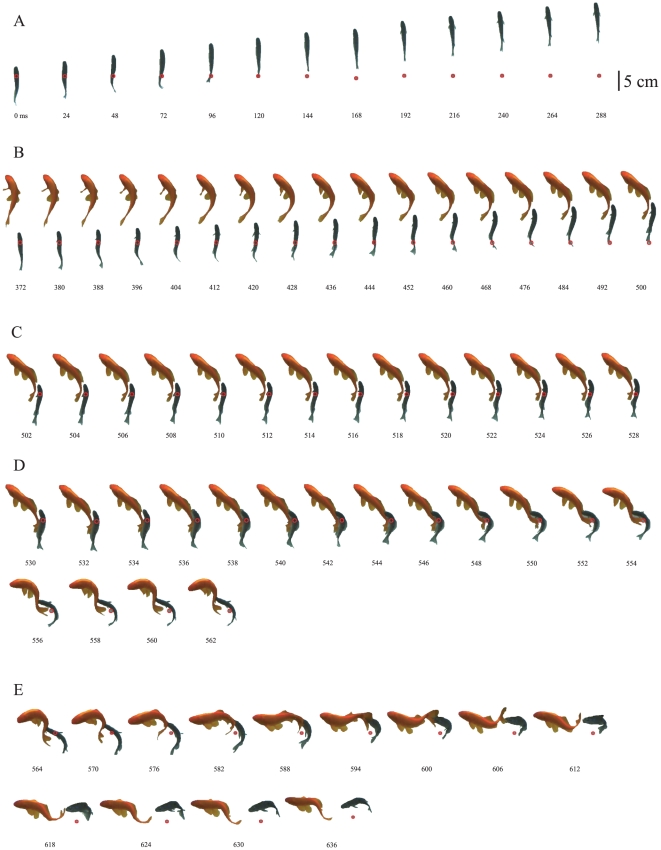
Behavioral sequence of *Perissodus microlepis* predation. A series of predation behaviors in a righty *P. microlepis* (black) consisted of the following five motions, by which scales were removed from a goldfish (red). (A) Approaching dash. (B) Stealthy swimming. (C) S-shaped posture. (D) Body flexion (J-bend). (E) Twisting (see details in text). Time in ms from the start of the approaching dash is denoted below the silhouettes. Red circles indicate the initial position of the fish during each subcomponent. See also Movie S1.

Typical predation was completed in approximately 600 ms and consisted of sequential behavioral subcomponents as follows: approaching the prey quickly from behind (370 ms; Figure 1A), moving stealthily to the preferred side of the prey (128 ms; Figure 1B), assuming an ‘S-shaped’ posture (26 ms; Figure 1C), striking the body of the prey with the mouth during quick body bending (32 ms; Figure 1D), and vertical twisting followed by releasing the mouth from the prey and closing the mouth (72 ms; Figure 1E). When a scale eater attacked a prey fish, it initially swam linearly toward its target from far behind the prey. After the scale eater was close to the prey, it moved stealthily to the side of the prey and then stopped to assume an S-shaped posture, probably by contracting the diagonal sides of the trunk muscles. Subsequently, the predator bent its body quickly into a J-shape and pressed a widely opened mouth onto the flank of the prey fish. In many cases, after biting at the prey fish's flank, the predator rapidly rotated its own body vertically to remove scales. Finally, it released the prey from its mouth. When dislodged scales remained floating in the water, the cichlid picked them up. An abrasion on the scales was observed on the prey after an attack.

In the 43 recorded approaches of a scale eater from behind its prey, as observed in the field, the scale eater exhibited vertical twisting in 24 cases, but finished the attack without twisting in the remaining cases. Thus the predation behavior of a scale eater consists of four or five subcomponents.

### Laterality of predation

**Figure 2:**
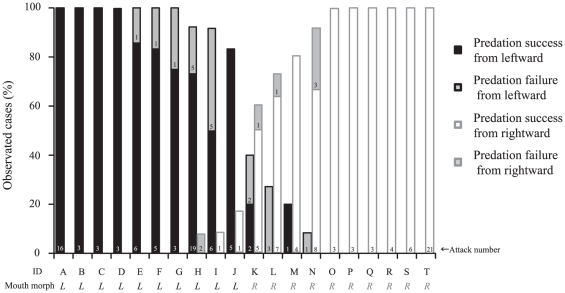
Percentage of left-side (black column) and right-side (open column) attacks for each predator (N = 20). Grey columns indicate failed attempts at scale eating. The numbers at the bottoms of the columns indicate the number of attacks by each fish. Asymmetric mouth morphology, lefty (*L*) or righty (*R*), is denoted for each fish. No lateral bias was observed in J, K, or L.

**Figure 3:**
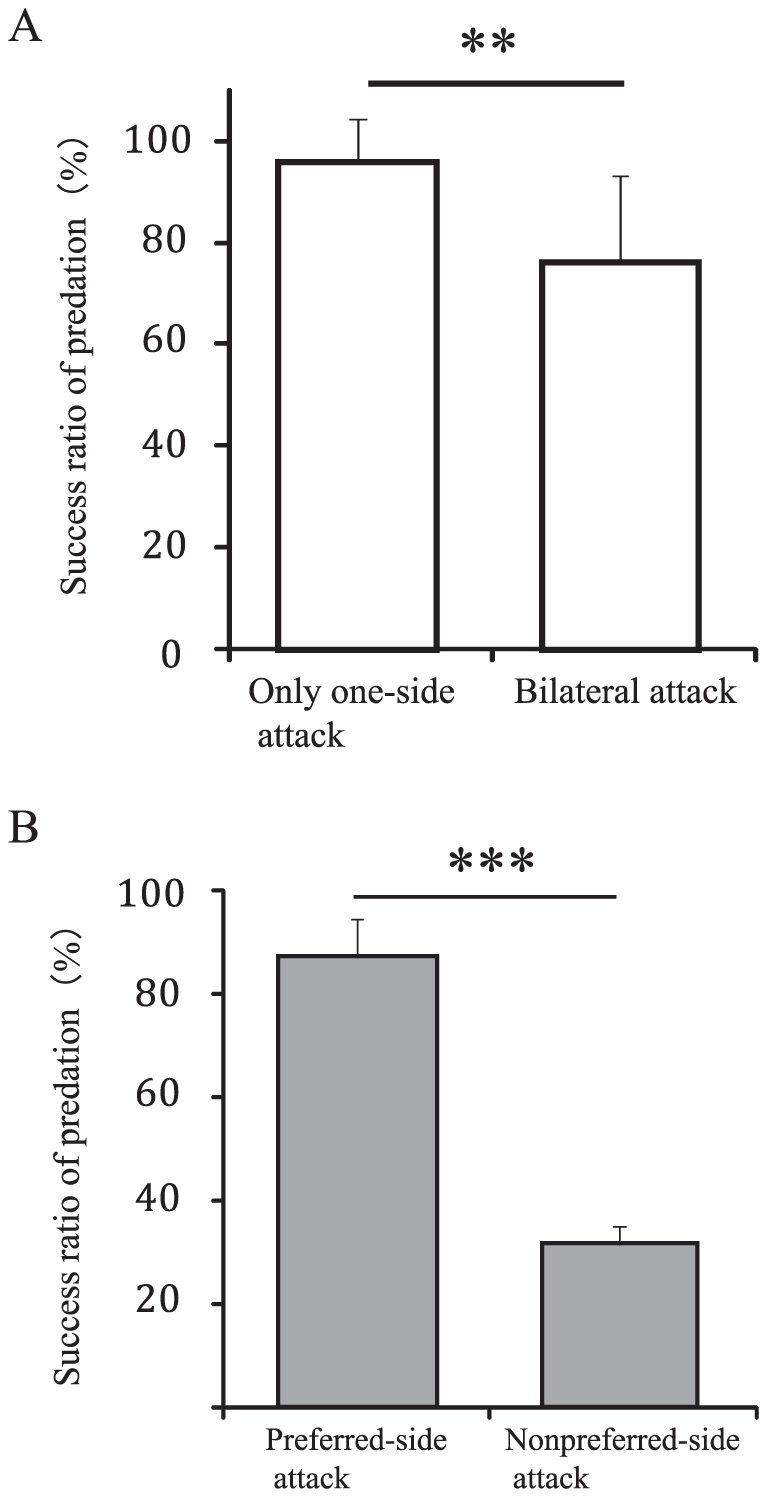
Comparison of predation success. (A) The success ratio of predation (mean ± SD) by fish that attacked only on one side (N = 13) and fish that attacked bilaterally (N = 7). (B) Weighted means of the predation success ratio for preferred- (N = 20) and nonpreferred-side attacks (N = 7). The weighted mean ratio is the value-weighted average of the ratios in which weight is proportional to the number of prey attacks. Error bars indicate 95% confidence intervals. P-values are from the Wilcoxon signed-rank test and GLMM analysis, respectively. **p<0.01, ***p<0.001.

Twenty individual *P. microlepis* attacked the prey goldfish 8.3±6.7 (± SD) times on average in an hour of observation. Most of the fish (18 of 20) exhibited a strong preference for attacking the prey on a specific side of the body in more than 80% of all trials (Figure 2), and 13 fish attacked only one side of the prey fishes' bodies. Eight of the 11 fish that made more than five attacks showed significant bias toward one attack side (binominal test: p<0.05). Predation success was significantly higher in fish that made only one-sided attacks than in those that attacked from both sides (Wilcoxon signed-rank test: χ^2^ = 6.962, p = 0.008; Figure 3A).

As previously reported, the *P. microlepis* in this study exhibited conspicuous asymmetry of mouth morphology (particularly in the lower jaws), corresponding to the direction of mouth opening [8]–[10], [14]. The mouths of all fish observed during the behavioral experiment opened either to the left side or to the right side: specifically, 10 fish were lefties and 10 were righties. We confirmed that the asymmetry index (AI) of the mouths of other individuals, calculated as the difference in height between the right and left mandible posterior ends, was clearly distributed bimodally (Figure S1C), and that the mouths of scale eaters with positive or negative AI values opened toward the left or toward the right, respectively.

The preferred attack side correlated significantly with mouth morphology (χ^2^ test: χ^2^ = 139.686, p<0.001; Figure 2): lefty fish, attacking prey preferentially from behind, moved to the left, flexed its body to the right and attacked the left flank of the prey, whereas righty fish flexed its body to the left to attack the right flank of the prey. The success ratio of attacks from the side on which the mouth opened (i.e., lefties attacking the left flank and vice versa) was significantly higher than that from the nonpreferred side (GLMM analysis: coefficient = 2.83, SEM = 0.74, z = 3.48, n = 20, p<0.001; Figure 3B).

### Kinematics of scale-eating behavior

**Figure 4:**
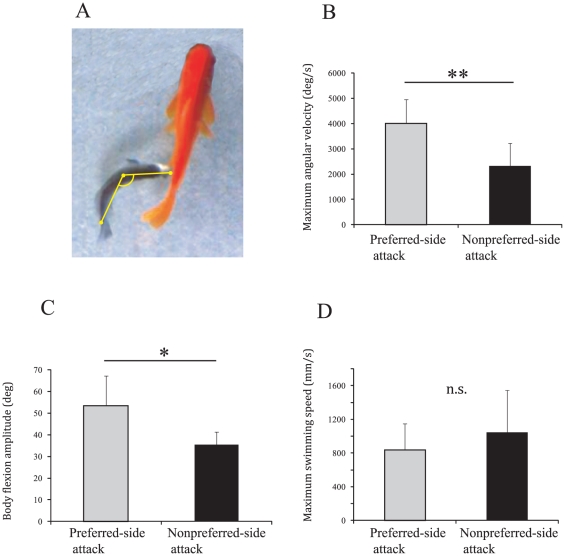
Kinematic differences in scale-eating behavior between preferred- and nonpreferred-side attacks. (A) The body flexion angle of scale-eating behavior was measured from three points on the body midline. (B) Maximum angular velocity (mean ± SD) of preferred-side (N = 39) and nonpreferred-side attacks (N = 4). (C) Body flexion amplitude. (D) Maximum swimming speed during the approach. P-values were obtained by means of the Wilcoxon signed-rank test. *p<0.05, **p<0.01, n.s., not significant.

Next, we analyzed the kinematics of 43 predation attacks that were clearly recorded with a high-speed video camera. A predation attack refers to a series of behaviors from chasing the prey fish to striking its flank. Each of these predation behaviors consisted of four or five behavioral components. Thirty-nine of the behaviors represented attacks from the preferred side, whereas the remainder were from the nonpreferred side. In preferred-side attacks, the maximum swimming speed during the approach phase ranged from 49 to 1475 mm s^−1^ (835±337, mean ± SD, n = 39). The maximum angular velocity and amplitude of body flexion were attained during the initial bending phase and ranged from 1584 to 5518 deg s^−1^ (4010±979) and from 24.3 to 88.5 deg (53.5±13.7), respectively. Notably, the maximum angular velocity was higher in preferred-side attacks than in nonpreferred-side attacks (Wilcoxon signed rank test: χ^2^ = 6.939, p = 0.008; Figure 4B). Similarly, the amplitude of body flexion was larger in preferred-side attacks than in nonpreferred-side attacks (χ^2^ = 6.294, p = 0.012; Figure 4C). Maximum swimming speed during approach, on the other hand, was not significantly different between attack sides (χ^2^ = 1.470, p = 0.225; Figure 4D).

### Startle response evoked by an acoustic perturbation

To compare body bends during scale-eating and escape, we examined the escape behavior of the same scale-eaters in response to a sound stimulus applied from below. Acoustically elicited startle responses in scale eaters were initiated with a typical C-shaped bend of the body at the initial phase of escape behavior, followed by a counter-bend and forward swimming, as previously reported in goldfish and zebrafish [16]–[18], [20]. The onset latency (from sound presentation to the onset of the C-shaped bend) was as short as that observed in goldfish (9.8±1.6 ms, mean ± SD, n = 63). During the startle response, the maximum angular velocity and amplitude of body flexion ranged from 4918 to 7696 deg s^−1^ (6484±634) and from 32.2 to 71.6 deg (56.4±9.1), respectively. The escape could be directed toward either side of the fish, exhibiting nonlateral bias (lefty: leftward bending vs. rightward bending = 18 vs. 19; χ^2^ test: χ^2^ = 0.027, p = 0.869; righty: 17 vs. 9, χ^2^ = 2.502, p = 0.114). Furthermore, no significant difference between leftward and rightward bending was observed in either maximum angular velocity (Wilcoxon signed-rank test: lefty, χ^2^ = 1.103, p = 0.294; righty, χ^2^ = 0.884, p = 0.347) or amplitude of body flexion (lefty, χ^2^ = 0.044, p = 0.834; righty, χ^2^ = 1.320, p = 0.251). Therefore, escape movements were neither correlated with mouth morphology nor laterally biased.

### Comparison between scale-eating and escape behavior

**Figure 5:**
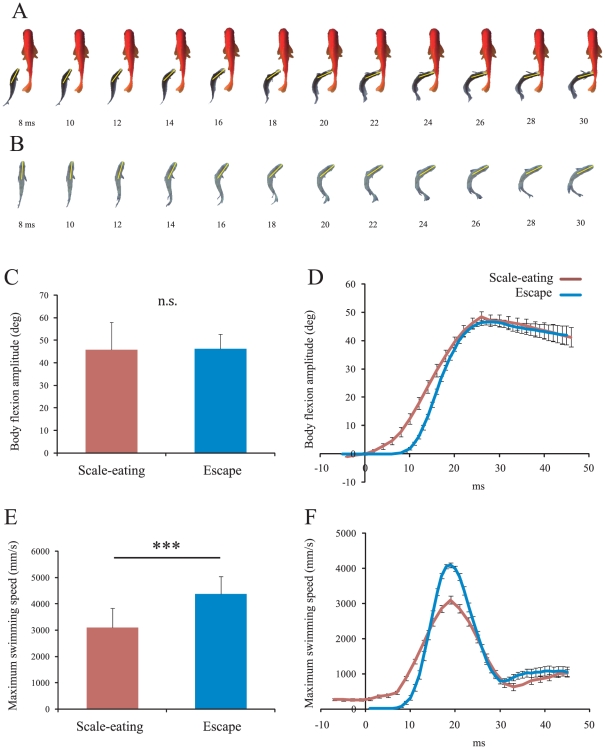
Comparison between scale-eating and escape behaviors. (A) Time-lapse images of *P. microlepis* in the bending phase of scale-eating. Yellow lines indicate the anterior midline from the snout to the center of mass. (B) An escape response elicited by acoustic stimulation consisted of a typical C-bend at the initial phase. During escape behavior, time zero was set at the time of acoustic stimulation, and maximum body flexion occurred at 26 ms. In order for the maximum body flexion angle in scale eating to occur at 26 ms after the acoustic stimulation (to match the time of occurrence of maximum body flexion in escape behavior), the time axis was adjusted. (C) The amplitude of the flexion angle (mean ± SD) of the anterior portion of the body during scale-eating (N = 39) and escape behaviors (N = 63). (D) Time course of change in the flexion angle (mean ± SE). (E) Maximum angular velocity of anterior body flexion. (F) Time course of the flexion angular velocity in scale-eating and escape behaviors. The maximum angular velocity occurred at 19 ms during escape and at 19 ms during scale eating. P-values were obtained by means of the Wilcoxon signed-rank test. **p<0.001, n.s., not significant.

Both scale-eating and escape behaviors include a fast lateral body flexion phase. During the scale-eating body flexion movement, the fish thrusts its mouth toward the prey and flexes the anterior part of the body into a “J-shape” while keeping the posterior part of the body straight (Figure 5A). During a fast escape, on the other hand, the whole body is bent into a C-shape (Figure 5B). We compared the kinematic elements of the body flexion between the two types of behaviors. In terms of the amplitude of anterior body flexion observed, there was no significant difference between scale eating and escape (Wilcoxon signed-rank test: χ^2^ = 0.003, p = 0.953; Figure 5C), whereas the maximum angular velocity of the body was significantly faster during escape than during scale eating (χ^2^ = 54.644, p<0.001; Figure 5E). Hence, we presume that the fast flexion of the anterior body during scale eating resembles that aspect of the C-bend during fast escape, though certain other movements, including mouth thrusts while keeping the posterior body straight, have apparently been added to the C-bend to create an effective posture for scale eating.

## Discussion

Monitoring *P. microlepis* scale-eating behavior in a tank revealed a remarkable laterality in the side and direction of attack and kinematics of the behavior. The preferred side of attack corresponded to the asymmetric mouth morphology, and higher predation success was achieved when direction of attack corresponded to asymmetric mouth morphology. Mouth morphology also correlated with a higher angular velocity of the body flexion in the direction of the prey during attack. Each predation behavior consisted of a series of up to five components: approaching the prey from behind, moving stealthily toward the prey's flank on the side of the predator's mouth opening, assuming an S-shaped posture, a quick body flexion (J-bend) to attack, and twisting to remove scales. In the field, prey fish take precautions against the approach of predators and are often able to escape in open water. Therefore, it is difficult for scale eater to success in predation (20% of all foraging attempts [21]). To overcome the difficulty, *P. microlepis* creeps up on its prey from behind because this offers it the protection of a blind spot [22]. The stealthily swimming from behind was also observed in the present experiment. In the subsequent stage, an extremely lateralized attack may require for providing a significant advantage at snatching scales from the prey flank.

Previous research on the stomach contents of *P. microlepis* has indirectly demonstrated that lefty fish attack only the left side of the prey flank and vice versa [9]. Lee et al. [23] reported that marks of dislodged scales were observed exclusively on one side of the prey's flank after an American cichlid prey fish was kept in a tank with a single *P. microlepis*. The side on which scales were dislodged coincided with the mouth-opening direction of the *P. microlepis*. Our study confirms these findings by presenting direct evidence of a one-to-one relationship between scale-eating behavior and mouth morphological phenotypes of *P. microlepis*.

Approximately 75% of *P. microlepis* individuals significantly preferred one side of the prey fish in the tank. In the case of the shrimp-eating cichlid *Neolamprologus fasciatus*, one-third of the observed individuals showed a significant lateral bias of the body when abutting on a rock while aiming at prey in the field [24]. Half of male Siamese fighting fish *Betta splendens* use a dominant eye to watch their own aggressive displays reflected in a mirror [25]. Thus the lateral behavioral preference of *P. microlepis* during scale eating is more conspicuous than the similar preferences seen in other lateralized fish. The above-mentioned species exhibit individual laterality with either a left- or right-bias. In contrast, behavioral laterality at the population level is observed in some fish: the majority of individuals in a population being lateralized in one direction [26]. For example, Australian lungfish mainly show C-start escape response to the left side yet turn to the right side during feeding behavior [27]. The interspecific difference of behavioral laterality remains a contentious matter.

The relationship between scale-eating behavior and mouth phenotype in scale eaters was closely associated with the kinematics of quick body flexion during an attack. Bending movements had significantly higher angular velocity and larger amplitude when the attack was made in the direction that the mouth faced. Consequently, a higher rate of success was achieved in attacks made on the side toward which the mouth opened. Thus the present study not only provides quantitative evidence that scale eaters base their direction of attack on their own mouth morphology but also reveals the presence of kinematic differences in attacks made from the two sides of a scale eater's body.

In addition to the asymmetric movement, we also first found that *P. microlepis* assumes an S-shaped posture just before attack bending. This specific posture seemed similar to that observed during the S-type escape (see [28] for a review) as well as to that observed during the feeding strikes of various other fish species (e.g., pike: [29], carp: [30], largemouth bass: [31], [32], zebrafish: [33], sculpin: [34]). The S-start response is formed by the diagonal activity between rostral and caudal body muscles [35]. The S-shaped posture has been suggested to result in a higher propulsive force during both escape and feeding [36]. Notably, this S-shaped posture in the scale eater was observed only during attacks from the preferred side. Therefore, the posture may be an indispensable preshape to achieving high body flexion speed during prey capture.

Anterior body flexion during an attack motion was similar to that used during escape. The similarity of body flexion exhibited during the two behaviors suggests that they may be elicited by shared neuronal networks, at least in part, as they both require nearly top speed. The high speed of body bending may lead to a higher survival rate of predation and escape. The kinematic parameters during both behaviors showed little fluctuation among individuals and trials, indicating that they are stereotypic behaviors. In teleosts, reticulospinal (RS) neurons play a key role producing large and fast body flexion during escape or feeding [37]. In our study, scale eaters exhibited a startle response to acoustic stimulation with a short onset latency of 5–11 ms and a C-shaped body bend at the initial phase. This startle response, known as the C-start, is identical to that observed in various teleosts when this behavior is initiated by the firing of one of the paired giant RS neurons, M-cells, in the hindbrain [16]–[18], [38]. The C-shaped profile is also observed in goldfish [39] and archer fish [40] during prey capture in which the firing of M-cells is shown or suspected to occur. Therefore, the M-cells may be involved in initiating the fast body bend during scale eating as during the escape response.

Some differences, however, were observed between the fast bend in scale eating and that in escape. In scale eating, fast bending occurred mainly in the anterior region of the body while keeping the posterior body straight and was associated with a mouth thrust, whereas the C-bend during escape was associated with simultaneous contraction of the trunk muscles on one side of the entire body without a mouth thrust. Thus, we cannot exclude the possibility that M-cells are not involved in the J-bend that occurs during scale eating. Nevertheless, the fast bend initiated by M-cells may be superposed with the preceding S-bend preshape. A combination of the S-shaped posture and the subsequently elicited C-bend may produce the J-bend, with bilateral contraction of the posterior trunk muscles. During J-bend, the posterior body may function as a pivot for strong body bending. If the lateralized performance is initiated by a lateral difference in the spinal cord and trunk muscles, the escape behavior should also have a lateral bias, as does the scale-eating behavior. However, no lateral bias was observed during the escape behavior of scale eaters for direction, maximum angular velocity, or body flexion angle. Thus, we suggest that the behavioral laterality in scale eating may be produced upstream of the common motor pathways.

Scale eaters obviously use visual cues at every stage of predation: in recognizing a prey fish, pursuing it, moving to its flank, and targeting its scales. A previous anatomical study has revealed projections from the tectum to the RS neurons involving the M-cells in zebrafish [41]. It has also been shown that MeLc and MeLr in RS neurons coordinate visually elicited prey capture movements [42] and that the ventromedial RS neurons (RoV3, MiV1, and MiV2) in the hindbrain perform an important role in visually eliciting turning movements [43]. In amphibians, pretectal pathways are necessary for encoding prey identity [44]. These observations suggest that the tectum and the RS neurons are involved in the visuomotor pathway controlling scale-eating behavior and that they are possible loci where the lateralized movements of scale eating are initiated. Additionally, mouth asymmetry in *P. microlepis* is minor in juveniles but becomes pronounced in adults [14]. It has been pointed out that the extent of mouth asymmetry in *P. microlepis* varies with foraging experience [45]. Thus, assessing whether scale-eating laterality is imposed during development through learning would be informative.

Behavioral laterality is believed to be associated with lateral differences in brain function, probably associated with structural asymmetry. However, our understanding of the neuronal mechanisms underlying behavioral laterality is largely rudimentary, and the outstanding questions have perplexed researchers for several decades [2], [4]. The system at work in scale eaters, which shows a clear functional laterality and consists of analyzable circuits, may disclose the relationship between behavior and brain lateralization.

## Materials and Methods

### Subjects

Large numbers of scale eaters (*Perissodus microlepis*) inhabit the rocky shore in the southern part of Lake Tanganyika [46]. In the field, the scale eater prefers to prey on *Tropheus moorei*, *Petrochromis* spp., *Ophthalmotilapia* spp., and *Lamprichthys tanganicanus* [22]. The scale eaters used for the behavioral experiment were collected from this lake (Cameron Bay, Zambia; 8°29′S, 30°27′E) and transported to Japan by a fish dealer. The fish were individually isolated in aquaria and maintained at 27°C, pH 8.3, and a 12 h∶12 h light∶dark cycle in the laboratory. Fish were fed pellets twice daily, except on the day before a predation experiment. These experiments started one month after the fish had been imported. Animal care of fish and all experimentation procedures were approved by the Institutional Animal Care Committee of Nagoya University (permit number #0014).

### Predation experiment

We used 20 scale eaters, each with a standard length of 82–101 mm (89.4±4.6, mean ± SD). For each iteration of the predation experiment, a *P. microlepis* and a prey goldfish (*Cyprinus carpio*; size 90–120 mm) were placed in a 90×45-cm tank with a water depth of approximately 15 cm. A brown plastic box was set up as a hiding space in the corner of the aquarium.

The dorsal view of predation was monitored with a FASTCAM high-speed video camera system (500 frames/s, 1024×1024 pixels, 1024PCI; Photron, Tokyo, Japan) positioned 1 m above the tank. The lateral view of predation behaviors was monitored simultaneously with a digital video camera (1920×1080 pixels, HDR-XR550V; Sony, Tokyo, Japan) positioned 1.5 m lateral of the tank and recording at 30 frames/s. The experimental tank was illuminated by three video camera lights (HVC-SL; Photron).

In the present study, goldfish were used as prey. Although *P. microlepis* does not encounter goldfish in Lake Tanganyika, the predation behavior of scale eaters appeared to be the same as those observed in the field [12], [22]. The scales lost by the prey fish were easily regenerated within 3 weeks [47].

Prior to each scale-eating behavior experiment, a scale eater was transferred to the experimental tank to habituate for 1 h. After a short time, it usually hid in the plastic box in the corner. One prey fish was then introduced into the opposite corner of the tank, and fish behavior was recorded using the cameras for up to 1 h. In terms of predation behavior, a “hit” occurred when the scale eater's mouth made contact with the flank of the prey fish, and a “miss” occurred when no such contact was made. Thereafter, both fish were moved back to their aquaria. In some cases, the movements of the scale eater were obscured because an event occurred out of frame or the images of the two fish overlapped. Only predation events that were clearly visible from the high-speed camera were used in subsequent analyses.

### Escape response experiment

Escape behavior in response to sound was tested in a cylindrical aquarium (diameter, 38 cm; water depth, 9 cm) that was surrounded by an obscure grey screen. Escape behaviors were monitored with a high-speed video camera (1000 frames/s) set above the aquarium. Acoustic stimulation (500 Hz, about 120 dB) was delivered from an underwater speaker (UW-30, University Sound; Electro-Voice, Buchanan, MI, USA) on the bottom of the aquarium, the driving voltage of which was fed by a function generator (DF1906; NF Corp., Tokyo, Japan).

Each scale eater was adapted to the aquarium for 1 h before the test. To avoid any confounding effects of the wall, we stimulated the fish only when it remained near the center of the aquarium. Because *P. microlepis* learned to habituate to a stimulus that was applied frequently, we applied five loud sound stimuli at intervals of more than 10 min.

### Kinematics of scale-eating and escape behaviors

The recorded images of scale-eating and escape behaviors were digitized with kinematic analysis software (Dipp-Motion 2D Pro; Direct Co. Ltd., Tokyo, Japan). Swimming speed (based on snout movement), body flexion angle, and angular velocity were measured. Body flexion angles were measured at three points on the midline of the body, as shown in figure 4A. These points were located at the snout, the caudal peduncle, and the center of mass [40], [48]. The longitudinal position of the center of mass was determined by laying a frozen stretched-straight fish (11.0±0.3 cm, total length, n = 5) on a balance so that the rostral and caudal body regions maintained equilibrium [35], [49]. The mean center of mass of the body was located at a relative distance of 38.3% from the snout (±2.0 SD). Angular velocity was calculated by dividing the change in the flexion angle observed in five sequential frames by the time. To compare the initial flexion of the anterior body in scale eating and escape, we measured the angular velocity along the shifted anterior midline from the snout to the center of mass during scale eating and escape, as well as during rest before escape and prior to a scale-eating attack.

### Assessment of the lateral difference in mouth morphology

*P. microlepis* exhibits dimorphic mouth asymmetry [8]–[10], [14], with mixed populations of ‘lefty’ and ‘righty’ individuals (Figure S1A). A lefty fish was identified by the following three characteristics (defined by [50]): the left lower jaw was clearly larger than the right one (Figure S1B), the left side of the head faced front, and the mouth opened rightward; a righty fish was identified by the opposite characteristics. An individual's mouth morphology as identified by these traits was always consistent [10]. The nature of this mouth asymmetry has been attributed to lateral differences in the length of the jaw joint [15]. After the behavioral experiments, the scale eaters were anesthetized in 0.01% eugenol and the mouth and craniofacial morphology were visually examined under a binocular microscope by two different researchers (Y.T. and Y.O.). All scale eaters used in the predation experiment were able to open their mouths wide in either direction.

Additionally, to assess the morphological asymmetry of the *P. microlepis* mouth, we measured the height of the mandible at the posterior end of the left and right lower jaws (called MPE height, see [10]; Figure S1B); this technique was applied to other fixed samples (n = 22) collected from Lake Tanganyika (Kasenga, 8°43′S, 31°08′E). The MPE height has been used to determine the size of the jaws, as it is similarly used to determine the length of the retroarticular process [51]. We took digital photographs and measured the MPE height using a digital microscope with image analysis software (VHX-100; Keyence, Osaka, Japan). The mandibles were independently positioned on the microscope for each of three replicate measurements to reduce observation errors. As the measurement of the posterior end of the mandible is prone to yielding some extreme values, median values, instead of mean values, were used for the following analysis. The measurement errors were small (ANOVA: *F*_43, 88_ = 998.80, p<0.001). Each fish's mouth opened toward the smaller side of its jaw. An index of asymmetry was calculated using the formula (height R – height L)×2×100/(height R+height L) [10], [24]. Positive values indicated a right-lower jaw that was larger than the left, and negative values indicated a left-lower jaw that was larger than the right. Fish with an index <0 were designated as lefty, and those with an index >0 were designated as righty (see [10]).

### Statistics

Significant individual preference for attacking a certain prey flank was determined by means of the binomial test (p<0.05). Individuals with low foraging motivation (total number of attacks <5 in 1 h) were omitted from the analysis. A generalized linear mixed model (GLMM) analysis was performed to ascertain the difference in predation success ratios between preferred- and nonpreferred-side attacks for each individual. We designed a GLMM with predation success (hit or miss) as the dependent variable and the following as independent variables: attack direction (preferred or nonpreferred side) as the fixed effect and individual as the random effect.

The GLMM analysis was performed using the R statistical package. Other statistical analyses were performed using JMP version 5 (SAS Institute Inc., Cary, NC, USA).

## Supporting Information

Figure S1 **Mouth asymmetry of** ***Perissodus microlepis*** **.** (A) Dorsal views of the mouth morphologies of lefty and righty fish. Yellow lines indicate the lateral tips of the lips. In the lefty fish, this line clearly leans to the right and vice versa. (B) The left and right lower jaws of a lefty fish. Arrow length represents the height of the mandible posterior end (MPE). The left-side jaw of this individual was larger than the right-side jaw. Scale bar = 5 mm. (C) Frequency distribution of the asymmetry index of the MPE height. A mouth morphology with a negative index denoted a lefty, and a positive index a righty. The frequency distribution was clearly bimodal and strongly deviated from normal (Shapiro–Wilk test: W = 0.756, p<0.001).
(TIF)

Movie S1 **Predation behavior of** ***Perissodus microlepis*** **(righty).** The first scene is at normal speed, and the subsequent scene is a slow playback (×0.06).
(MPG)
