# Supplementary figures and images for: Lateralized Kinematics of Predation Behavior in a Lake Tanganyika Scale-Eating Cichlid Fish
Source: PLoS One. 2012 Jan 6;7(1):e29272. doi: 10.1371/journal.pone.0029272 (PMC3253053; doi:10.1371/journal.pone.0029272)

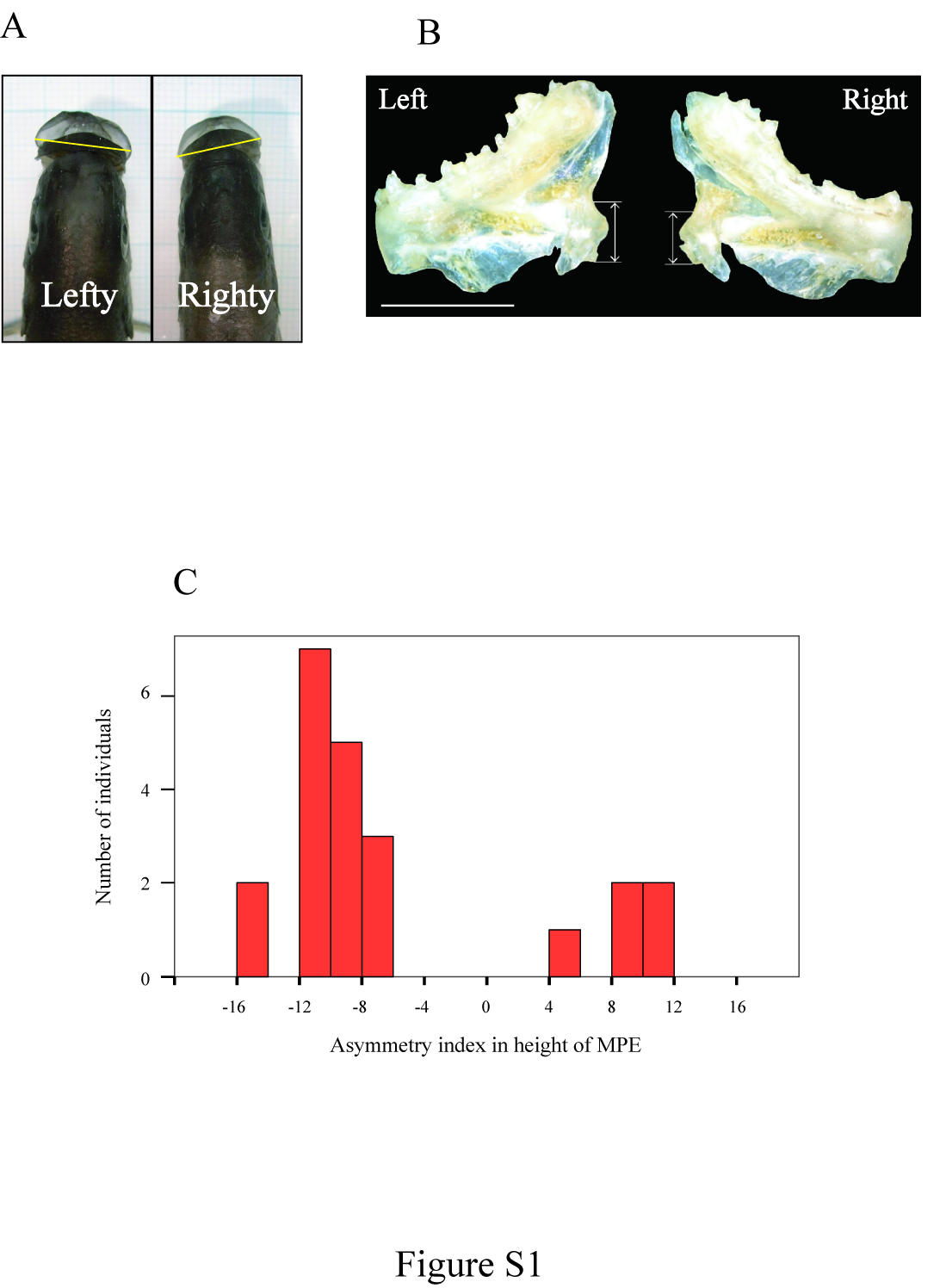

Supplement: Figure S1 — Mouth asymmetry of Perissodus microlepis . (A) Dorsal views of the mouth morphologies of lefty and righty fish. Yellow lines indicate the lateral tips of the lips. In the lefty fish, this line clearly leans to the right and vice versa. (B) The left and right lower jaws of a lefty fish. Arrow length represents the height of the mandible posterior end (MPE). The left-side jaw of this individual was larger than the right-side jaw. Scale bar = 5 mm. (C) Frequency distribution of the asymmetry index of the MPE height. A mouth morphology with a negative index denoted a lefty, and a positive index a righty. The frequency distribution was clearly bimodal and strongly deviated from normal (Shapiro–Wilk test: W = 0.756, p<0.001). (TIF) [file pone.0029272.s001.tif]
